# Crystal structure of bis(4-benzoyl­pyridine-κ*N*)bis(methanol-κ*O*)bis(thio­cyanato-κ*N*)nickel(II) methanol monosolvate

**DOI:** 10.1107/S2056989019001555

**Published:** 2019-01-31

**Authors:** Carsten Wellm, Christian Näther

**Affiliations:** a Institut für Anorganische Chemie, Universität Kiel, Max-Eyth. Str. 2, 241128 Kiel

**Keywords:** crystal structure, nickel(II) thio­cyanate, discrete complex, solvate, hydrogen bonding

## Abstract

The asymmetric unit of the title compound, [Ni(NCS)_2_(C_12_H_9_NO)_2_(CH_3_OH)_2_]·CH_3_OH, comprises one Ni^II^ cation, two thio­cyanate anions, two 4-benzoyl­pyridine coligands, two coordinating, as well as one non-coordinating methanol mol­ecule. The Ni^II^ cation is coordinated by two terminally N-bonded thio­cyanate anions, the N atoms of two 4-benzoyl­pyridine coligands and the O atoms of two methanol ligands within a slightly distorted octa­hedron. Individual complexes are linked by inter­molecular O—H⋯S hydrogen bonding into chains parallel to [010] that are further connected into layers parallel to (10) by C—H⋯S hydrogen bonds. Additional C—H⋯O hydrogen-bonding inter­actions lead to the formation of a three-dimensional network that limits channels extending parallel to [010] in which the non-coordinating methanol mol­ecules are located. They are hydrogen-bonded to the coordinating methanol mol­ecules. X-ray powder diffraction revealed that the compound could not be prepared as a pure phase.

## Abstract 2

In the crystal structure of the title compound, the Ni^II^ cations are octa­hedrally coordinated by two terminally N-bonded thio­cyanate anions, two methanol mol­ecules and two 4-benzoyl­pyridine coligands into discrete complexes that are linked by inter­molecular C—H⋯S, C—H⋯O and O—H⋯O hydrogen-bonding inter­actions into a three-dimensional network with channels in which the non-coordinating methanol solvate mol­ecules are located.

## Chemical context   

Thio­cyanate anions are versatile ligands that can coordinate to metal cations in different manners, leading to a variety of structural set-ups. The most common coordination modes are the N-terminal and the *μ*-1,3-bridging coordination, but, as an example, there are also reports of a *μ*-1,1-coordination (Prananto *et al.*, 2017 ▸; Buckingham, 1994 ▸; Palion-Gazda *et al.*, 2017 ▸; Mautner *et al.*, 2016 ▸, 2017 ▸; Mahmoudi *et al.*, 2017 ▸; Hamdani *et al.*, 2018 ▸; Wöhlert *et al.*, 2014*a* ▸,*b* ▸). With respect to paramagnetic transition metal cations, the *μ-*1,3-bridging mode is of special importance because it can mediate the magnetic exchange (Gonzalez *et al.*, 2012 ▸; Wöhlert *et al.*, 2013**a* ▸,b* ▸; Palion-Gazda *et al.*, 2015 ▸; Guillet *et al.*, 2016 ▸; Mekuimemba *et al.*, 2018 ▸). In this context, an increasing number of compounds with different magnetic properties are being reported (Wöhlert *et al.*, 2014**a* ▸,b* ▸; Werner *et al.*, 2015 ▸; Suckert *et al.*, 2017*a* ▸; Mautner *et al.*, 2018 ▸). In the majority of cases, the metal cations are linked by thio­cyanate anions into chains, but there are also examples where layer formation is observed (Suckert *et al.*, 2016 ▸; Wellm *et al.*, 2018 ▸; Neumann *et al.*, 2018*a* ▸).

Unfortunately, for most paramagnetic transition metal cations, the bridging modes are energetically less favored and thus, compounds with a terminal coordination are usually obtained. Nevertheless, we have found an alternative approach to overcome this problem by transformation of the latter compounds through thermal annealing into the desired compounds that have bridging anions. For the alternative synthesis of such coordination polymers with bridging anionic ligands, a precursor consisting of a discrete complex can be used in which the metal cations are coordinated by two terminal N-bonded thio­cyanate anions and four co-ligands that, in our cases, consist of pyridine derivatives. Upon controlled heating, two of the four co-ligands can be removed. Frequently, this treatment yields the desired compounds with bridging coordination as inter­mediates, which can easily be investigated by thermogravimetry. In some cases, no discrete decomposition steps are observed because all co-ligands are removed in one step. Under these circumstances, alternatives are required that are based on precursor complexes comprising only two of the pyridine derivatives as ligands and two coordinating and volatile mol­ecules such as water or methanol. The ligand mol­ecules are emitted in a discrete step (also observable in a thermogravimetrical measurement), which directly produces the desired compounds in qu­anti­tative yield. It is also noted that this approach often leads to the formation of polymorphs or isomers that are different from the compounds obtained from solution (Werner *et al.*, 2015 ▸; Jochim *et al.*, 2018 ▸).
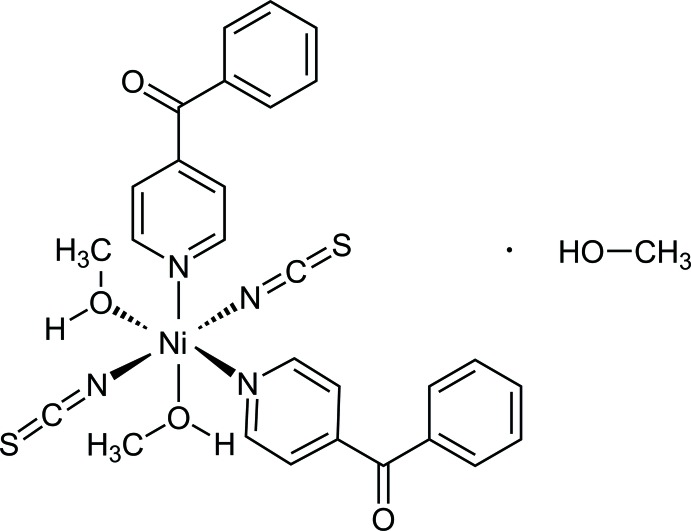


In this context we have reported two isotypic compounds with chain-structures that have the general composition *M*(NCS)_2_(4-benzoyl­pyridine)_2_ where *M* = Co, Ni (Rams *et al.*, 2017 ▸; Jochim *et al.*, 2018 ▸). Here the metal cations are linked into linear chains with a *cis*–*cis*–*trans* coordination, in contrast to most other compounds with similar linear chains where all ligands are in *trans* positions. This is somewhat surprising because Cd(NCS)_2_(4-benzoyl­pyridine)_2_ also forms linear chains with an all-*trans* coordination of the cations (Neumann *et al.*, 2018*a* ▸). Therefore, our intention was to test if a different isomer with, for example, Ni can be prepared by thermal annealing. A complex with composition Ni(NCS)_2_(4-benzoyl­pyridine)_4_ has already been reported in the literature. It decomposes in several steps, but only the inter­mediate after complete removal of 4-benzoyl­pyridine was examined (Soliman *et al.*, 2014 ▸). We have synthesized this compound again and investigated its thermal properties. The residue formed after removal of half of the 4-benzoyl­pyiridine ligands is of poor crystallinity and does not correspond to a pure phase. Therefore, we searched for a more promising precursor; during these investigations, crystals of the title compound were obtained and characterized by single crystal X-ray diffraction. X-ray powder diffraction revealed that the compound directly isolated from the reaction mixture is a nearly pure phase but always contaminated with a very small amount of Ni(NCS)_2_(4-benzoyl­pyridine)_4_ (see Fig. S1 in the supporting information). More importantly, if the title compound is filtered off, it decomposes very quickly into an unknown crystalline phase that does not correspond to that of Ni(NCS)_2_(4-benzoyl­pyridine)_4_ already reported in the literature. However, this sample is still contaminated with Ni(NCS)_2_(4-benzoyl­pyridine)_4_, and any attempt to completely index its powder pattern failed (Fig. S2 in the supporting information).

## Structural commentary   

**Figure 1:**
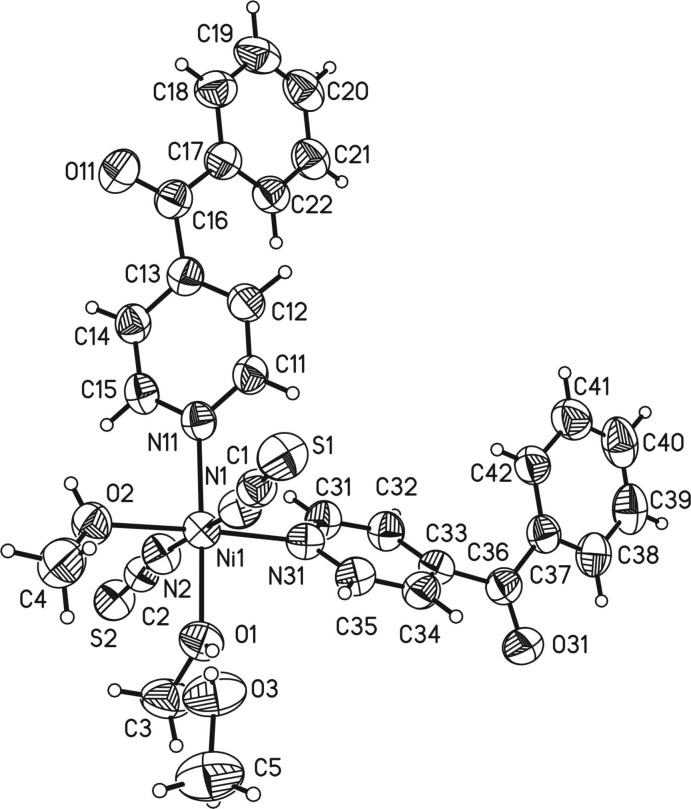
The asymmetric unit of the solvated title complex with the atom labelling and displacement ellipsoids drawn at the 50% probability level.

The crystal structure of the title compound consists of discrete complexes in which the Ni^II^ cations are sixfold coordinated by two crystallographically independent thio­cyanate anions, two methanol mol­ecules and two 4-benzoyl­pyridine ligands (Fig. 1 ▸). The Ni—N bond lengths to the anionic ligands of 2.009 (3) and 2.034 (3) Å are shorter than those to the 4-benzoyl­pyridine ligands [2.092 (2) and 2.104 (2) Å; the Ni—O distances to the methanol ligands are longer again at 2.108 (2) and 2.154 (2) Å. The coordination sphere around Ni^II^ can be described as a slightly distorted octa­hedron. This is also obvious from the angle variance and the quadratic elongation, which were calculated to be 4.7 and 1.022 (Robinson *et al.*, 1971 ▸). The 4-benzoyl­pyridine ligand is not planar. The dihedral angle between the pyridine ring (N11, C11–C15) and the carbonyl plane (C13, C16, C17, O11) amounts to 56.86 (16)° and that between the phenyl ring (C17–C22) and the carbonyl group (C13, C16, C17, O11) to 12.49 (17)°. The second ligand has corresponding values of 48.61 (17)° between the pyridine ring (N31, C31–C35) and the carbonyl group (C33, C36, C37, O31) and 16.69 (18)° between the phenyl ring (C37–C42) and the carbonyl group (C33, C36, C37, O31). There is a short intra­molecular contact between one of the aromatic hydrogen atoms (H35) and one of the thio­cyanate N atoms (N1); however, the corresponding C—H⋯N angle deviates strongly from linearity, indicating only a weak inter­action (Table 1 ▸).

## Supra­molecular features   

**Figure 2:**
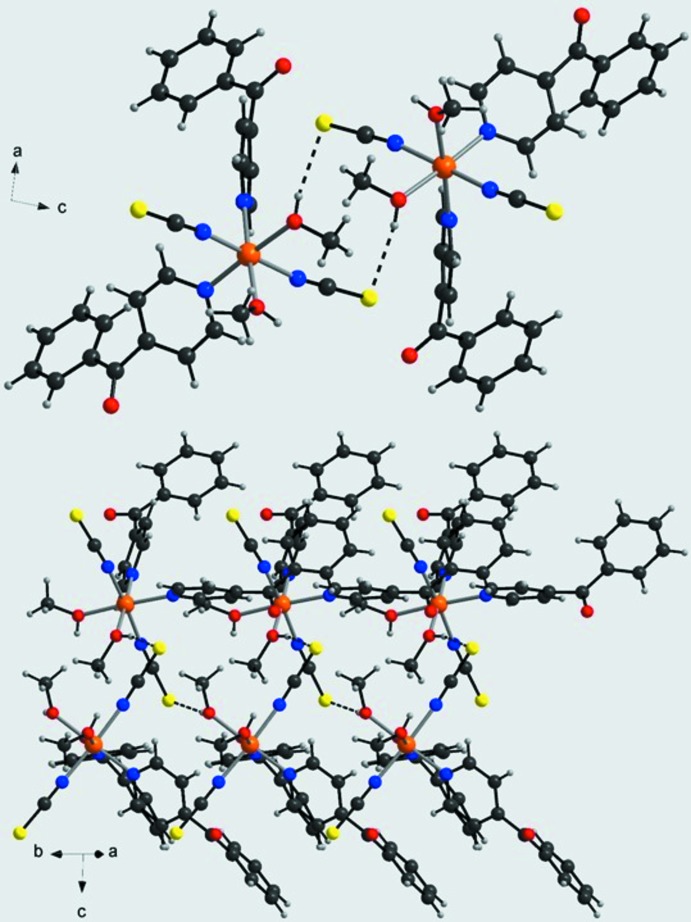
Crystal structure of the title compound in a view along (top) and perpendicular (bottom) to the hydrogen-bonded chains. Inter­molecular O—H⋯S hydrogen bonding is shown as dashed lines.

**Figure 3:**
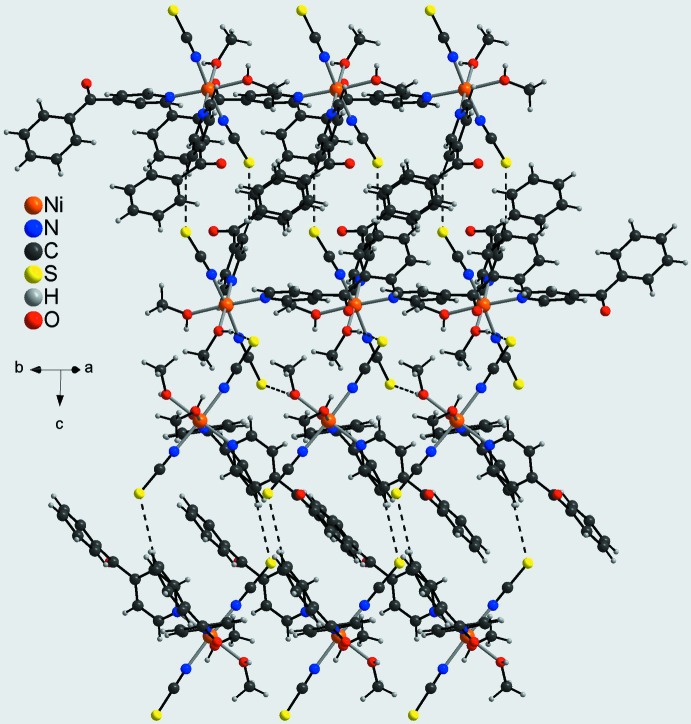
Crystal structure of the title compound in a view approximately [110] showing the layers formed by inter­molecular O—H⋯S and C—H⋯S hydrogen bonding (shown as dashed lines).

**Figure 4:**
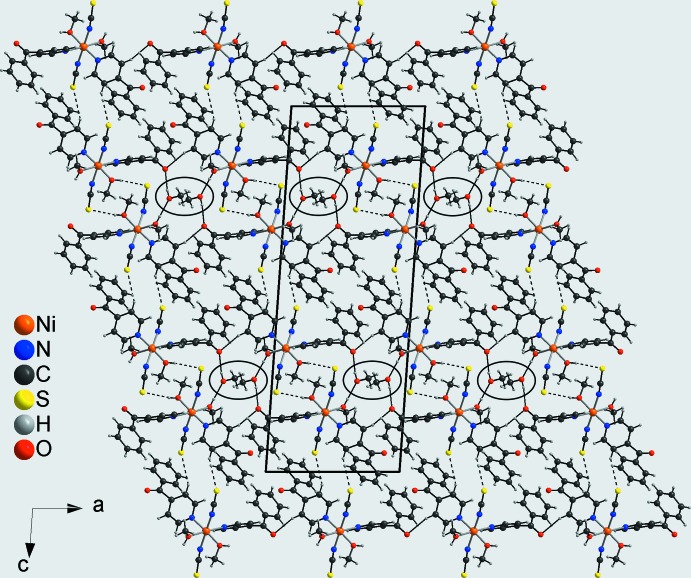
Crystal structure of the title compound in a view along [010] showing the channels that are filled with the non-coordinating methanol mol­ecules. Inter­molecular O—H⋯S, C—H⋯S, C—H⋯O and O—H⋯O hydrogen bonding is shown as dashed lines; the oval channels are marked with thick lines.

The crystal structure of the title compound is dominated by extensive inter­molecular classical and non-classical hydrogen-bonding inter­actions of medium-to-weak strengths (Table 1 ▸). Discrete complexes are linked by inter­molecular O—H⋯S hydrogen bonds into chains extending parallel to [010] (Fig. 2 ▸, top). Within such a chain, the complexes are related by the 2_1_-screw axis, resulting in a helical arrangement (Fig. 2 ▸, bottom). These chains are further linked by pairs of centrosymmetric C—H⋯S hydrogen bonds into layers extending parallel to (10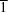) (Fig. 3 ▸). Adjacent layers are linked into a three-dimensional network by C—H⋯O hydrogen bonding between a hydrogen atom (H34) of one of the phenyl rings and the carbonyl O atom (O11) of a neighboring 4-benzoyl­pyridine ligand (Fig. 4 ▸). Within this network channels are formed in which the non-coordinating methanol mol­ecules are embedded (Fig. 4 ▸). The solvent mol­ecules are linked by O—H⋯O hydrogen bonding and act both as a donor (O3) to a neighbouring carbonyl O atom (O11) and as an acceptor for a hydroxyl group (O1) of a methanol ligand (Fig. 4 ▸).

## Database survey   

In the Cambridge Structure Database (Version 5.39, last update Aug 2018; Groom *et al.*, 2016 ▸) several structures of transition-metal thio­cyanate coordination compounds with 4-benzoyl­pyridine as ligand have been deposited. They include three compounds with the composition [*M*(NCS)_2_(4-benzoyl­pyridine)_2_]_*n*_ (*M* = Cd, Co, Ni), in which the metal cations are octa­hedrally coordinated and linked into chains by pairs of *μ*-1,3 bridging thio­cyanate anions (Neumann *et al.*, 2018**a* ▸;* Rams *et al.*, 2017 ▸; Jochim *et al.*, 2018 ▸). Discrete complexes with general composition *M*(NCS)_2_(4-benzoyl­pyridine)_4_ (*M* = Co, Ni, Mn, Cd and Zn) are also reported in which the metal cations are octa­hedrally coordinated by two terminal N-bonded thio­cyanate anions and four 4-benzoyl­pyridine co-ligands (Drew *et al.*, 1985 ▸; Soliman *et al.*, 2014 ▸; Wellm & Näther, 2018 ▸; Neumann *et al.*, 2018*b* ▸). There are also compounds where the metal cations are fourfold coordinated by the two N-bonded terminal thio­cyanate anions and two 4-benzoyl­pyridine co-ligands, forming either a tetra­hedral (Zn^II^ complex) or a square-planar (Cu^II^ complex) coordination sphere (Neumann *et al.*, 2018*a* ▸; Bai *et al.*, 2011 ▸). The last group consists of octa­hedrally coordinated Co^II^ cations that either contain two aceto­nitrile (Suckert *et al.*, 2017*b* ▸) or two methanol mol­ecules (Suckert *et al.*, 2017*c* ▸) as coordinating solvent mol­ecules.

## Synthesis and crystallization   

Ba(SCN)_2_·3H_2_O and 4-benzoyl­pyridine were purchased from Alfa Aesar. NiSO_4_·6H_2_O was purchased from Merck. All solvents and reactants were used without further purification. Ni(NCS)_2_ was prepared by the reaction of equimolar amounts of NiSO_4_·6H_2_O and Ba(NCS)_2_·3H_2_O in water. The resulting white precipitate of BaSO_4_ was filtered off, and the solvent was evaporated from the filtrate. The product was dried at room temperature. Crystals of the title compound suitable for single-crystal X-ray diffraction were obtained within a few days by the reaction of 52.5 mg Ni(NCS)_2_ (0.30 mmol) with 27.5 mg 4-benzoyl­pyridine (0.15 mmol) in methanol (1.5 ml) at 354 K using culture tubes.

## Refinement   

Crystal data, data collection and structure refinement details are summarized in Table 2 ▸. The hydrogen atoms were positioned with idealized geometry (C–H = 0.95–0.98 Å; methyl H atoms in part were allowed to rotate but not to tip) and were refined with *U*_iso_(H) = 1.2*U*_eq_(C) (1.5 for methyl H atoms) using a riding model. The OH hydrogen atoms were located in a difference-Fourier map; their bond lengths were set to ideal values and finally they were refined with *U*_iso_(H) = 1.5*U*_eq_(O) using a riding model.

## Supplementary Material

Crystal structure: contains datablock(s) I. DOI: 10.1107/S2056989019001555/wm5485sup1.cif

Structure factors: contains datablock(s) I. DOI: 10.1107/S2056989019001555/wm5485Isup2.hkl

Figure S1. Calculated X-ray powder pattern of the title compound (A) and of [Ni(NCS)2(4-benzoylpyridine)4] (B) as well as the experimental X-ray powder pattern (C) of the freshly prepared title compound isolated before filtration.. DOI: 10.1107/S2056989019001555/wm5485sup3.tif

Figure S2. Calculated X-ray powder pattern of the title compound (A), of [Ni(NCS)2(4-benzoylpyridine)4] (B) and [Ni(NCS)2(4-benzoylpyridine)2] (C) as well as the experimental X-ray powder pattern (D) of the title compound isolated after filtration with a Buchner funnel.. DOI: 10.1107/S2056989019001555/wm5485sup4.tif

CCDC reference: 1894170

Additional supporting information:  crystallographic information; 3D view; checkCIF report

## Figures and Tables

**Table 1:** Hydrogen-bond geometry (Å, °)

*D*—H⋯*A*	*D*—H	H⋯*A*	*D*⋯*A*	*D*—H⋯*A*
C32—H32⋯S2^i^	0.95	3.01	3.865 (3)	151
C34—H34⋯O11^ii^	0.95	2.50	3.406 (4)	160
C35—H35⋯N1	0.95	2.65	3.113 (4)	111
O1—H1⋯O3	0.84	1.83	2.643 (3)	163
O2—H2⋯S1^iii^	0.84	2.44	3.246 (2)	160
O3—H3⋯O11^iii^	0.84	1.98	2.808 (3)	166

Symmetry codes: (i) 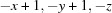; (ii) 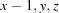; (iii) 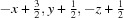.

**Table 2:** Experimental details

Crystal data	
Chemical formula	[Ni(NCS)_2_(C_12_H_9_NO)_2_(CH_4_O)_2_]·CH_4_O
*M*_r_	637.40
Crystal system, space group	Monoclinic, *P*2_1_/*n*
Temperature (K)	200
*a*, *b*, *c* (Å)	12.0588 (6), 7.5515 (3), 33.0408 (16)
β (°)	94.021 (4)
*V* (Å^3^)	3001.4 (2)
*Z*	4
Radiation type	Mo *K*α
μ (mm^−1^)	0.83
Crystal size (mm)	0.25 × 0.15 × 0.08

Data collection	
Diffractometer	Stoe *IPDS2*
Absorption correction	Numerical (*X-SHAPE* and *X-RED32*; Stoe, 2008 ▸)
*T*_min_, *T*_max_	0.638, 0.875
No. of measured, independent and observed [*I* > 2σ(*I*)] reflections	17675, 4726, 4004
*R*_int_	0.036
θ_max_ (°)	24.1
(sin θ/λ)_max_ (Å^−1^)	0.574

Refinement	
*R*[*F* ^2^ > 2σ(*F* ^2^)], *wR*(*F* ^2^), *S*	0.041, 0.105, 1.10
No. of reflections	4726
No. of parameters	371
H-atom treatment	H-atom parameters constrained
Δρ_max_, Δρ_min_ (e Å^−3^)	0.46, −0.26

Computer programs: *X-AREA* (Stoe, 2008 ▸), *XP* in *SHELXTL* and *SHELXS97* (Sheldrick, 2008 ▸), *SHELXL2014*/7 (Sheldrick, 2015 ▸), *DIAMOND* (Brandenburg, 1999 ▸) and *publCIF* (Westrip, 2010 ▸).

## supplementary crystallographic information

### Crystal data

**Table:**

[Ni(NCS)_2_(C_12_H_9_NO)_2_(CH_4_O)_2_]·CH_4_O	*F*(000) = 1328
*M**_r_* = 637.40	*D*_x_ = 1.411 Mg m^−^^3^
Monoclinic, *P*2_1_/*n*	Mo *K*α radiation, λ = 0.71073 Å
*a* = 12.0588 (6) Å	Cell parameters from 17675 reflections
*b* = 7.5515 (3) Å	θ = 1.2–24.1°
*c* = 33.0408 (16) Å	µ = 0.83 mm^−^^1^
β = 94.021 (4)°	*T* = 200 K
*V* = 3001.4 (2) Å^3^	Block, green
*Z* = 4	0.25 × 0.15 × 0.08 mm

### Data collection

**Table:**

Stoe IPDS-2 diffractometer	4004 reflections with *I* > 2σ(*I*)
ω scans	*R*_int_ = 0.036
Absorption correction: numerical (*X-SHAPE* and *X-RED32*; Stoe, 2008)	θ_max_ = 24.1°, θ_min_ = 1.2°
*T*_min_ = 0.638, *T*_max_ = 0.875	*h* = −13→13
17675 measured reflections	*k* = −8→8
4726 independent reflections	*l* = −37→37

### Refinement

**Table:**

Refinement on *F*^2^	0 restraints
Least-squares matrix: full	Hydrogen site location: mixed
*R*[*F*^2^ > 2σ(*F*^2^)] = 0.041	H-atom parameters constrained
*wR*(*F*^2^) = 0.105	*w* = 1/[σ^2^(*F*_o_^2^) + (0.0571*P*)^2^ + 0.7722*P*] where *P* = (*F*_o_^2^ + 2*F*_c_^2^)/3
*S* = 1.10	(Δ/σ)_max_ = 0.001
4726 reflections	Δρ_max_ = 0.46 e Å^−^^3^
371 parameters	Δρ_min_ = −0.26 e Å^−^^3^

### Special details

**Table:**

Geometry. All esds (except the esd in the dihedral angle between two l.s. planes) are estimated using the full covariance matrix. The cell esds are taken into account individually in the estimation of esds in distances, angles and torsion angles; correlations between esds in cell parameters are only used when they are defined by crystal symmetry. An approximate (isotropic) treatment of cell esds is used for estimating esds involving l.s. planes.

### Fractional atomic coordinates and isotropic or equivalent isotropic displacement parameters (Å^2^)

**Table:**

	*x*	*y*	*z*	*U*_iso_*/*U*_eq_	
Ni1	0.58556 (3)	0.48583 (5)	0.16271 (2)	0.04735 (13)	
N1	0.5502 (2)	0.3421 (3)	0.21226 (7)	0.0550 (6)	
C1	0.5445 (2)	0.2585 (4)	0.24141 (9)	0.0488 (7)	
S1	0.53684 (8)	0.13868 (12)	0.28229 (2)	0.0659 (2)	
N2	0.6201 (2)	0.6432 (3)	0.11620 (8)	0.0584 (6)	
C2	0.6290 (2)	0.7419 (4)	0.08932 (8)	0.0488 (6)	
S2	0.64295 (7)	0.87918 (12)	0.05253 (2)	0.0638 (2)	
N11	0.7197 (2)	0.3188 (3)	0.15150 (7)	0.0487 (5)	
C11	0.7138 (3)	0.1421 (4)	0.15605 (9)	0.0528 (7)	
H11	0.6443	0.0913	0.1614	0.063*	
C12	0.8037 (3)	0.0310 (4)	0.15339 (9)	0.0545 (7)	
H12	0.7950	−0.0936	0.1556	0.065*	
C13	0.9062 (2)	0.1032 (4)	0.14754 (8)	0.0516 (7)	
C14	0.9127 (3)	0.2864 (4)	0.14232 (9)	0.0534 (7)	
H14	0.9819	0.3406	0.1378	0.064*	
C15	0.8191 (3)	0.3869 (4)	0.14378 (8)	0.0524 (7)	
H15	0.8244	0.5107	0.1391	0.063*	
C16	1.0109 (3)	−0.0051 (4)	0.14785 (9)	0.0543 (7)	
C17	1.0192 (3)	−0.1532 (4)	0.11893 (9)	0.0538 (7)	
C18	1.1107 (3)	−0.2650 (5)	0.12354 (11)	0.0645 (8)	
H18	1.1639	−0.2506	0.1459	0.077*	
C19	1.1240 (3)	−0.3976 (5)	0.09531 (12)	0.0723 (10)	
H19	1.1861	−0.4751	0.0985	0.087*	
C20	1.0476 (3)	−0.4176 (4)	0.06269 (11)	0.0689 (9)	
H20	1.0577	−0.5080	0.0433	0.083*	
C21	0.9566 (3)	−0.3071 (4)	0.05800 (10)	0.0638 (8)	
H21	0.9038	−0.3216	0.0355	0.077*	
C22	0.9424 (3)	−0.1748 (4)	0.08619 (9)	0.0555 (7)	
H22	0.8797	−0.0986	0.0830	0.067*	
O11	1.08903 (18)	0.0371 (3)	0.17175 (7)	0.0681 (6)	
N31	0.4723 (2)	0.3367 (3)	0.12595 (7)	0.0496 (6)	
C31	0.4886 (3)	0.2922 (4)	0.08771 (9)	0.0536 (7)	
H31	0.5575	0.3230	0.0773	0.064*	
C32	0.4118 (3)	0.2047 (4)	0.06254 (9)	0.0553 (7)	
H32	0.4283	0.1743	0.0357	0.066*	
C33	0.3094 (2)	0.1610 (4)	0.07678 (9)	0.0510 (7)	
C34	0.2925 (3)	0.2062 (4)	0.11660 (9)	0.0541 (7)	
H34	0.2241	0.1780	0.1277	0.065*	
C35	0.3740 (3)	0.2911 (4)	0.13971 (9)	0.0534 (7)	
H35	0.3608	0.3194	0.1670	0.064*	
C36	0.2167 (3)	0.0776 (4)	0.05069 (9)	0.0556 (7)	
C37	0.2378 (3)	−0.0814 (4)	0.02630 (9)	0.0529 (7)	
C38	0.1594 (3)	−0.1303 (5)	−0.00466 (9)	0.0626 (8)	
H38	0.0944	−0.0608	−0.0100	0.075*	
C39	0.1759 (4)	−0.2788 (5)	−0.02748 (11)	0.0747 (10)	
H39	0.1228	−0.3105	−0.0488	0.090*	
C40	0.2696 (4)	−0.3824 (5)	−0.01951 (12)	0.0828 (12)	
H40	0.2800	−0.4858	−0.0352	0.099*	
C41	0.3480 (3)	−0.3361 (5)	0.01117 (12)	0.0758 (10)	
H41	0.4120	−0.4077	0.0167	0.091*	
C42	0.3325 (3)	−0.1839 (4)	0.03387 (10)	0.0596 (8)	
H42	0.3869	−0.1501	0.0546	0.071*	
O31	0.1247 (2)	0.1446 (3)	0.05102 (8)	0.0762 (7)	
O1	0.45471 (18)	0.6547 (3)	0.17699 (6)	0.0590 (5)	
H1	0.4265	0.6291	0.1988	0.089*	
C3	0.4305 (3)	0.8282 (5)	0.16255 (13)	0.0801 (11)	
H3A	0.3642	0.8727	0.1747	0.120*	
H3B	0.4937	0.9062	0.1700	0.120*	
H3C	0.4171	0.8256	0.1330	0.120*	
O2	0.69448 (18)	0.6461 (3)	0.20203 (6)	0.0604 (5)	
H2	0.7622	0.6195	0.2026	0.091*	
C4	0.6653 (3)	0.7267 (6)	0.23878 (12)	0.0854 (12)	
H4A	0.7293	0.7924	0.2510	0.128*	
H4B	0.6030	0.8082	0.2330	0.128*	
H4C	0.6437	0.6349	0.2577	0.128*	
O3	0.3644 (2)	0.6458 (4)	0.24757 (8)	0.0844 (8)	
H3	0.3838	0.5988	0.2700	0.127*	
C5	0.2807 (4)	0.7704 (7)	0.25548 (14)	0.1051 (15)	
H5A	0.2179	0.7090	0.2666	0.158*	
H5B	0.2553	0.8303	0.2302	0.158*	
H5C	0.3109	0.8580	0.2752	0.158*	

### Atomic displacement parameters (Å^2^)

**Table:**

	*U*^11^	*U*^22^	*U*^33^	*U*^12^	*U*^13^	*U*^23^
Ni1	0.0524 (2)	0.0467 (2)	0.0431 (2)	−0.00002 (16)	0.00477 (15)	0.00073 (15)
N1	0.0544 (15)	0.0607 (15)	0.0499 (13)	0.0041 (12)	0.0036 (11)	0.0030 (12)
C1	0.0465 (16)	0.0523 (16)	0.0477 (15)	0.0025 (13)	0.0045 (12)	−0.0028 (13)
S1	0.0660 (5)	0.0740 (5)	0.0582 (4)	0.0013 (4)	0.0086 (4)	0.0192 (4)
N2	0.0653 (17)	0.0552 (15)	0.0551 (14)	−0.0033 (12)	0.0074 (12)	−0.0009 (12)
C2	0.0482 (16)	0.0511 (16)	0.0471 (15)	−0.0007 (13)	0.0036 (13)	−0.0006 (13)
S2	0.0660 (5)	0.0682 (5)	0.0574 (4)	−0.0027 (4)	0.0056 (4)	0.0168 (4)
N11	0.0527 (14)	0.0484 (13)	0.0454 (12)	−0.0049 (11)	0.0066 (11)	−0.0028 (10)
C11	0.0473 (16)	0.0508 (17)	0.0609 (17)	−0.0061 (13)	0.0089 (14)	−0.0011 (13)
C12	0.0552 (18)	0.0482 (16)	0.0607 (17)	−0.0008 (14)	0.0076 (14)	−0.0016 (13)
C13	0.0521 (17)	0.0562 (17)	0.0468 (14)	−0.0021 (14)	0.0064 (13)	−0.0059 (13)
C14	0.0509 (17)	0.0540 (17)	0.0566 (16)	−0.0069 (14)	0.0129 (14)	−0.0041 (13)
C15	0.0595 (18)	0.0475 (15)	0.0508 (15)	−0.0096 (14)	0.0091 (14)	−0.0030 (12)
C16	0.0503 (17)	0.0591 (18)	0.0542 (16)	−0.0020 (14)	0.0084 (14)	0.0003 (14)
C17	0.0517 (17)	0.0541 (17)	0.0568 (17)	−0.0014 (14)	0.0114 (14)	0.0025 (13)
C18	0.0559 (19)	0.065 (2)	0.074 (2)	0.0064 (16)	0.0109 (16)	0.0085 (16)
C19	0.074 (2)	0.0584 (19)	0.087 (2)	0.0152 (17)	0.029 (2)	0.0090 (18)
C20	0.082 (2)	0.0527 (18)	0.075 (2)	0.0000 (17)	0.028 (2)	−0.0030 (16)
C21	0.072 (2)	0.0563 (18)	0.0642 (19)	−0.0028 (17)	0.0143 (16)	−0.0047 (15)
C22	0.0573 (18)	0.0527 (17)	0.0571 (16)	0.0004 (14)	0.0092 (14)	−0.0001 (13)
O11	0.0538 (13)	0.0825 (16)	0.0674 (13)	−0.0034 (11)	0.0007 (12)	−0.0082 (12)
N31	0.0513 (14)	0.0516 (13)	0.0464 (12)	0.0024 (11)	0.0056 (11)	0.0013 (10)
C31	0.0535 (17)	0.0590 (17)	0.0492 (15)	−0.0048 (14)	0.0096 (13)	−0.0017 (13)
C32	0.0594 (19)	0.0586 (17)	0.0481 (15)	−0.0037 (15)	0.0053 (14)	−0.0052 (13)
C33	0.0511 (17)	0.0450 (15)	0.0566 (16)	0.0026 (13)	0.0021 (14)	0.0010 (13)
C34	0.0506 (17)	0.0531 (17)	0.0591 (17)	0.0005 (14)	0.0084 (14)	0.0016 (14)
C35	0.0545 (18)	0.0576 (17)	0.0486 (15)	0.0014 (14)	0.0070 (14)	0.0013 (13)
C36	0.0528 (19)	0.0538 (17)	0.0594 (17)	−0.0007 (14)	−0.0019 (14)	0.0020 (14)
C37	0.0575 (18)	0.0497 (16)	0.0520 (16)	−0.0070 (14)	0.0083 (14)	0.0016 (13)
C38	0.067 (2)	0.068 (2)	0.0534 (17)	−0.0148 (17)	0.0042 (15)	0.0026 (15)
C39	0.085 (3)	0.080 (2)	0.0602 (19)	−0.029 (2)	0.0132 (18)	−0.0120 (18)
C40	0.096 (3)	0.073 (2)	0.084 (3)	−0.027 (2)	0.037 (2)	−0.025 (2)
C41	0.076 (2)	0.062 (2)	0.092 (3)	−0.0005 (18)	0.025 (2)	−0.0069 (19)
C42	0.0594 (19)	0.0527 (17)	0.0674 (19)	−0.0043 (15)	0.0101 (15)	−0.0008 (15)
O31	0.0551 (14)	0.0694 (15)	0.1020 (18)	0.0078 (12)	−0.0103 (13)	−0.0142 (13)
O1	0.0676 (14)	0.0550 (12)	0.0551 (11)	0.0074 (10)	0.0087 (10)	0.0036 (9)
C3	0.080 (3)	0.063 (2)	0.099 (3)	0.0178 (19)	0.021 (2)	0.023 (2)
O2	0.0602 (13)	0.0646 (13)	0.0567 (11)	−0.0027 (10)	0.0057 (10)	−0.0121 (10)
C4	0.069 (2)	0.111 (3)	0.076 (2)	0.004 (2)	0.0030 (19)	−0.042 (2)
O3	0.0767 (17)	0.108 (2)	0.0711 (15)	0.0280 (15)	0.0213 (13)	0.0161 (14)
C5	0.095 (3)	0.129 (4)	0.092 (3)	0.033 (3)	0.018 (3)	−0.002 (3)

### Geometric parameters (Å, º)

**Table:**

Ni1—N2	2.009 (3)	C31—H31	0.9500
Ni1—N1	2.034 (3)	C32—C33	1.392 (4)
Ni1—N31	2.092 (2)	C32—H32	0.9500
Ni1—N11	2.104 (2)	C33—C34	1.388 (4)
Ni1—O1	2.108 (2)	C33—C36	1.501 (4)
Ni1—O2	2.154 (2)	C34—C35	1.362 (4)
N1—C1	1.158 (4)	C34—H34	0.9500
C1—S1	1.634 (3)	C35—H35	0.9500
N2—C2	1.170 (4)	C36—O31	1.220 (4)
C2—S2	1.615 (3)	C36—C37	1.478 (4)
N11—C11	1.345 (4)	C37—C42	1.387 (5)
N11—C15	1.345 (4)	C37—C38	1.394 (4)
C11—C12	1.378 (4)	C38—C39	1.373 (5)
C11—H11	0.9500	C38—H38	0.9500
C12—C13	1.378 (4)	C39—C40	1.384 (6)
C12—H12	0.9500	C39—H39	0.9500
C13—C14	1.397 (4)	C40—C41	1.382 (6)
C13—C16	1.503 (4)	C40—H40	0.9500
C14—C15	1.364 (4)	C41—C42	1.393 (5)
C14—H14	0.9500	C41—H41	0.9500
C15—H15	0.9500	C42—H42	0.9500
C16—O11	1.228 (4)	O1—C3	1.417 (4)
C16—C17	1.479 (4)	O1—H1	0.8401
C17—C22	1.383 (4)	C3—H3A	0.9800
C17—C18	1.389 (5)	C3—H3B	0.9800
C18—C19	1.386 (5)	C3—H3C	0.9800
C18—H18	0.9500	O2—C4	1.424 (4)
C19—C20	1.376 (5)	O2—H2	0.8400
C19—H19	0.9500	C4—H4A	0.9800
C20—C21	1.380 (5)	C4—H4B	0.9800
C20—H20	0.9500	C4—H4C	0.9800
C21—C22	1.384 (4)	O3—C5	1.418 (5)
C21—H21	0.9500	O3—H3	0.8402
C22—H22	0.9500	C5—H5A	0.9800
N31—C31	1.335 (4)	C5—H5B	0.9800
N31—C35	1.343 (4)	C5—H5C	0.9800
C31—C32	1.369 (4)		
			
N2—Ni1—N1	175.96 (10)	N31—C31—C32	123.9 (3)
N2—Ni1—N31	92.09 (10)	N31—C31—H31	118.0
N1—Ni1—N31	90.85 (10)	C32—C31—H31	118.0
N2—Ni1—N11	90.95 (10)	C31—C32—C33	119.1 (3)
N1—Ni1—N11	91.66 (9)	C31—C32—H32	120.4
N31—Ni1—N11	93.10 (9)	C33—C32—H32	120.4
N2—Ni1—O1	90.70 (10)	C34—C33—C32	117.1 (3)
N1—Ni1—O1	86.56 (9)	C34—C33—C36	119.6 (3)
N31—Ni1—O1	89.25 (9)	C32—C33—C36	123.2 (3)
N11—Ni1—O1	177.08 (8)	C35—C34—C33	119.9 (3)
N2—Ni1—O2	88.77 (10)	C35—C34—H34	120.0
N1—Ni1—O2	88.15 (9)	C33—C34—H34	120.0
N31—Ni1—O2	176.81 (9)	N31—C35—C34	123.4 (3)
N11—Ni1—O2	89.96 (9)	N31—C35—H35	118.3
O1—Ni1—O2	87.67 (8)	C34—C35—H35	118.3
C1—N1—Ni1	171.3 (2)	O31—C36—C37	122.2 (3)
N1—C1—S1	179.4 (3)	O31—C36—C33	117.5 (3)
C2—N2—Ni1	172.8 (3)	C37—C36—C33	120.2 (3)
N2—C2—S2	179.2 (3)	C42—C37—C38	119.5 (3)
C11—N11—C15	117.0 (3)	C42—C37—C36	121.7 (3)
C11—N11—Ni1	121.87 (19)	C38—C37—C36	118.8 (3)
C15—N11—Ni1	120.7 (2)	C39—C38—C37	120.2 (4)
N11—C11—C12	123.3 (3)	C39—C38—H38	119.9
N11—C11—H11	118.3	C37—C38—H38	119.9
C12—C11—H11	118.3	C38—C39—C40	120.3 (4)
C13—C12—C11	119.1 (3)	C38—C39—H39	119.8
C13—C12—H12	120.5	C40—C39—H39	119.8
C11—C12—H12	120.5	C41—C40—C39	120.3 (4)
C12—C13—C14	117.9 (3)	C41—C40—H40	119.9
C12—C13—C16	123.0 (3)	C39—C40—H40	119.9
C14—C13—C16	119.0 (3)	C40—C41—C42	119.5 (4)
C15—C14—C13	119.5 (3)	C40—C41—H41	120.2
C15—C14—H14	120.2	C42—C41—H41	120.2
C13—C14—H14	120.2	C37—C42—C41	120.2 (3)
N11—C15—C14	123.0 (3)	C37—C42—H42	119.9
N11—C15—H15	118.5	C41—C42—H42	119.9
C14—C15—H15	118.5	C3—O1—Ni1	128.6 (2)
O11—C16—C17	121.9 (3)	C3—O1—H1	114.5
O11—C16—C13	118.0 (3)	Ni1—O1—H1	114.0
C17—C16—C13	120.1 (3)	O1—C3—H3A	109.5
C22—C17—C18	119.8 (3)	O1—C3—H3B	109.5
C22—C17—C16	121.5 (3)	H3A—C3—H3B	109.5
C18—C17—C16	118.6 (3)	O1—C3—H3C	109.5
C19—C18—C17	119.6 (3)	H3A—C3—H3C	109.5
C19—C18—H18	120.2	H3B—C3—H3C	109.5
C17—C18—H18	120.2	C4—O2—Ni1	125.3 (2)
C20—C19—C18	120.3 (3)	C4—O2—H2	112.4
C20—C19—H19	119.9	Ni1—O2—H2	115.5
C18—C19—H19	119.9	O2—C4—H4A	109.5
C19—C20—C21	120.3 (3)	O2—C4—H4B	109.5
C19—C20—H20	119.8	H4A—C4—H4B	109.5
C21—C20—H20	119.8	O2—C4—H4C	109.5
C20—C21—C22	119.7 (3)	H4A—C4—H4C	109.5
C20—C21—H21	120.1	H4B—C4—H4C	109.5
C22—C21—H21	120.1	C5—O3—H3	106.0
C17—C22—C21	120.2 (3)	O3—C5—H5A	109.5
C17—C22—H22	119.9	O3—C5—H5B	109.5
C21—C22—H22	119.9	H5A—C5—H5B	109.5
C31—N31—C35	116.6 (3)	O3—C5—H5C	109.5
C31—N31—Ni1	123.5 (2)	H5A—C5—H5C	109.5
C35—N31—Ni1	119.82 (19)	H5B—C5—H5C	109.5

### Hydrogen-bond geometry (Å, º)

**Table:**

*D*—H···*A*	*D*—H	H···*A*	*D*···*A*	*D*—H···*A*
C32—H32···S2^i^	0.95	3.01	3.865 (3)	151
C34—H34···O11^ii^	0.95	2.50	3.406 (4)	160
C35—H35···N1	0.95	2.65	3.113 (4)	111
O1—H1···O3	0.84	1.83	2.643 (3)	163
O2—H2···S1^iii^	0.84	2.44	3.246 (2)	160
O3—H3···O11^iii^	0.84	1.98	2.808 (3)	166

Symmetry codes: (i) −*x*+1, −*y*+1, −*z*; (ii) *x*−1, *y*, *z*; (iii) −*x*+3/2, *y*+1/2, −*z*+1/2.
